# Repetition-Based Approach for Task Adaptation in Imitation Learning

**DOI:** 10.3390/s22186959

**Published:** 2022-09-14

**Authors:** Tho Nguyen Duc, Chanh Minh Tran, Nguyen Gia Bach, Phan Xuan Tan, Eiji Kamioka

**Affiliations:** 1 Graduate School of Engineering and Science, Shibaura Institute of Technology, Tokyo 135-8548, Japan; 2 Department of Information and Communications Engineering, Shibaura Institute of Technology, Tokyo 135-8548, Japan

**Keywords:** imitation learning, task adaptation, repetition learning, transfer learning, generative adversarial network

## Abstract

Transfer learning is an effective approach for adapting an autonomous agent to a new target task by transferring knowledge learned from the previously learned source task. The major problem with traditional transfer learning is that it only focuses on optimizing learning performance on the target task. Thus, the performance on the target task may be improved in exchange for the deterioration of the source task’s performance, resulting in an agent that is not able to revisit the earlier task. Therefore, transfer learning methods are still far from being comparable with the learning capability of humans, as humans can perform well on both source and new target tasks. In order to address this limitation, a task adaptation method for imitation learning is proposed in this paper. Being inspired by the idea of repetition learning in neuroscience, the proposed adaptation method enables the agent to repeatedly review the learned knowledge of the source task, while learning the new knowledge of the target task. This ensures that the learning performance on the target task is high, while the deterioration of the learning performance on the source task is small. A comprehensive evaluation over several simulated tasks with varying difficulty levels shows that the proposed method can provide high and consistent performance on both source and target tasks, outperforming existing transfer learning methods.

## 1. Introduction

Reinforcement learning (RL) is an effective method to solve sequential decision-making tasks, where a learning agent interacts with the environment to improve its performance through trial and error [1]. RL has achieved exceptional success in challenging tasks, such as object manipulation [2,3,4,5], game playing [6,7,8,9], and autonomous driving [10,11,12,13]. Despite its remarkable advancement, RL still faces appealing difficulties caused by the need of a reward function [14,15]. For each task that the agent has to accomplish, a carefully designed reward function must be provided. However, designing a hand-crafted reward function may require too much time or expense, especially in complex tasks. This problem has motivated a number of research studies on imitation learning (IL), where expert-generated demonstration data are provided instead of a reward function in order to help the agent learn how to perform a task [16,17]. For this reason, IL has been growing in popularity and achieved some successes in numerous tasks, including robotics control [18,19,20] and autonomous driving [21,22,23,24].

Despite certain achievements, IL agents are designed to focus on accomplishing only a single, narrowly defined task. Therefore, when given a new task, the agent has to start the learning process again from the ground up, even if it has already learned a task that is related to and shares the same structure with the new one. On the other hand, humans possess an astonishing ability in the learning process, where the knowledge learned from source tasks can be leveraged for learning a new task. For example, an infant can reuse and augment the motor skills obtained when he learns to walk or uses his hand, for more complex tasks later in his life (e.g., riding a bike). Transfer learning (TL) is a technique based on this idea. TL enables the agent to reuse its knowledge learned from a source task in order to facilitate learning a new target task, resulting in a more generalized agent.

Recent studies have applied TL to RL/IL agents and achieved some success, especially in robot manipulation tasks since these tasks usually share a common structure (i.e., robot arm) [25,26,27]. Nevertheless, there is still an enormous difference between human ability and TL. Since TL is designed to leverage the learned knowledge to accelerate the acquisition of the new target task, the learning performance on the target task may be improved in exchange for the deterioration of the source task’s performance. In other words, the agent forgets how to perform the previously learned task when learning a new one, which is described as the catastrophic forgetting problem [28,29]. On the contrary, humans can perform well on both source and target tasks.

To address the aforementioned gap, a novel challenge on task adaptation in imitation learning is discussed in this paper, in which a trained agent on a source task faces a new target task and must optimize its overall performance on both tasks. In order words, the research objective is to help the agent achieve high learning performance on the target task, while avoiding the performance deterioration on the source task. The problem can be served as a step toward building a general-purpose agent. As one illustrative example, consider a household robot learning to assist its human owner. Initially, the human might want to teach the robot to load clothes into the washer by providing demonstrations of the task. At a later time, the user could teach the robot to fold clothes. These tasks are related to each other since they involve manipulating clothes, hence the robot is expected to perform well on both tasks and leverage any relevant knowledge obtained from loading the washer while folding clothes. In order to achieve such a knowledge transfer ability, a task adaptation method for imitation learning is proposed in this paper. Being inspired by the idea of repetition learning in neuroscience [30,31,32], the general idea of the proposed method is to make the agent repeatedly review the learned knowledge of the source task while learning the target task at the same time. Accordingly, the proposed method is two-fold. Firstly, to allow the agent to repeatedly review the learned knowledge of the source task, a task adaptation algorithm is proposed. In the adaptation process, the learned knowledge is expanded by adding the knowledge of the target task. Secondly, a novel IL agent which is capable of finding an optimal policy using expert-generated demonstrations, is proposed. This agent allows the learned knowledge of the source task to be encoded into a high-dimensional vector, namely task embedding, which then supports the knowledge expansion in the adaptation process. The evaluation results show that the proposed method has a better learning ability compared to existing transfer learning approaches.

The main contributions of this work are summarized as follows:

- An imitation learning agent is proposed to learn an optimal policy using expert-generated demonstration data. The agent is capable of encoding its knowledge into high-dimensional task embedding space in order to support the knowledge expansion in the later adaptation process.
- Given a new target task, a task adaptation algorithm is proposed in order to enable the agent to broaden its knowledge without forgetting the previous source task by leveraging the idea of repetition learning in neuroscience. The resulting agent can provide a better generalization and consistently perform well on both source and target tasks.
- A set of experiments are conducted over a number of simulated tasks in order to evaluate the performance of the proposed task adaptation method in terms of success rate, average cumulative reward, and computational cost. The evaluation results demonstrate the effectiveness of the proposed method in comparison with existing transfer learning methods.

The rest of the paper is organized as follows: Section 2 reviews existing studies on transfer learning and some existing works that are related to the proposed method. The formulation of the task adaptation problem in imitation learning is presented in Section 3. A detailed description of the proposed approach is provided in Section 4. Section 5 provides the details of the experimental settings and results. Section 6 discusses the potential of the proposed method in real-world problems. The conclusion is given in Section 7.

## 2. Related Work

Transfer learning (TL) aims to accelerate, adapt, and improve the agent’s learning process on a new target task by transferring knowledge learned from the previous source task. Whereas TL has been intensively studied and shown appealing performance in supervised learning [33,34,35,36,37,38,39], it remains an open question in reinforcement learning and imitation learning fields. Fine tuning is the most explored approach for transfer learning in both RL and IL settings [40,41,42]. In fine tuning, the RL/IL agent is pre-trained on a source task and then retrained to a new target task. Fine tuning does not require strong assumptions about the target domain, making it an easily applicable approach. There are different approaches to transfer learning that have been proposed, such as reward shaping [43,44,45], inter-task mapping [46,47,48], representation learning [49,50,51], etc. However, these methods were designed for RL agents; directly applying them to transfer an IL agent does not necessarily lead to successful results since RL and IL differ in many factors. Moreover, the key challenge in transfer learning is catastrophic forgetting, in which the agent tends to unexpectedly lose the knowledge that was learned from the source task while transferring to the new target task. The reason is due to the changes in the agent’s network parameters that are related to the source task getting overwritten to fulfill the target task’s objectives [28]. Therefore, TL methods are not suitable for an agent that revisits the earlier task. In contrast, instead of transferring the knowledge learned from the source task to a new target task, the proposed adaptation method attempts to expand the agent’s learned knowledge. The knowledge expansion allows the agent to learn a new target task while retaining the previously learned source task’s knowledge, resulting in an agent that can perform well on both the source and target tasks after adaptation.

Besides transfer learning, the proposed adaptation method of learning to perform both source and target tasks also bears similarity to multi-task learning, where an agent is trained to perform multiple tasks simultaneously [52,53,54,55,56]. In multi-task learning, the knowledge transfer is enabled by learning a shared representation among tasks. However, in this study, the proposed adaptation method focuses on learning the source and target tasks sequentially. In addition, the performance deterioration on the previously learned source task is more highlighted compared to both transfer learning and multi-task learning.

## 3. Problem Formulation

The task adaptation problem in IL can be formalized as a sequential Markov decision process (MDP). A MDP $M_{x}$ for a task *x* with finite time horizon $H_{x}$ [1] is represented as the following equation:

(1) $$M_{x}=\left(S_{x},A_{x},P_{x},R_{x},\gamma_{x},H_{x}\right)$$

where $S_{x}$ and $A_{x}$ represent the continuous state and action spaces, respectively; $P_{x}:S_{x}\times A_{x}\times S_{x}\to R^{+}$ denotes the transition probability function; $R_{x}:S_{x}\times A_{x}\to R$ is the reward function; and $\gamma_{x}\in \left(0,1\right]$ is the discount factor. In the IL setting, the reward function $R_{x}$ is unknown. A stochastic policy $\pi_{x}:S_{x}\to P\left(A_{x}\right)$ for $M_{x}$ describes a mapping from each state to the probability of taking each action. The goal of an IL agent is to learn an optimal policy $\pi_{x}^{*}$ that imitates the expert policy ${\hat{\pi }}_{x}$ given demonstrations from that expert. An expert demonstration for a task *x* is defined as a sequence of state–action pairs $\tau_{x}=\left\{\left({\hat{s}}_{x}^{t},{\hat{a}}_{x}^{t}\right):t\in \left[0,H_{x}\right]\right\}$.

Let $M_{S}$ denote a source task, which provides prior knowledge $K_{S}$ that is accessible by the target task $M_{T}$, such that by leveraging $K_{S}$, the target agent learns better in the target task $M_{T}$. The main objective in this study is to learn an optimal policy $\pi_{ST}^{*}\left(K_{S},K_{T}\right)$ for both source and target tasks, by leveraging $K_{T}$ from $M_{T}$ as well as $K_{S}$ from $M_{S}$.

## 4. The Proposed Agent and Adaptation Algorithm

The proposed method presented in this section involves two main processes: learning from a source task and adapting to a new target task. The main objective is to build an agent that can perform consistently well on both source and target tasks. In order to achieve this, the general of this novel idea is to allow the agent to repeatedly review the knowledge learned from the source task, while learning the new knowledge of the target task. The idea is inspired by a human learning effect, which is repetition learning. Prior studies in neuroscience have proved that when humans learn by repetition, their memory performance can be enhanced and retained for a longer time [30,31,32], giving humans the unique ability to perform most sophisticated tasks with ease. Therefore, in this paper, developing a similarly intelligent method is focused on in order to achieve the main research objective and to tackle the task adaptation problem in imitation learning.

**Figure 1:**
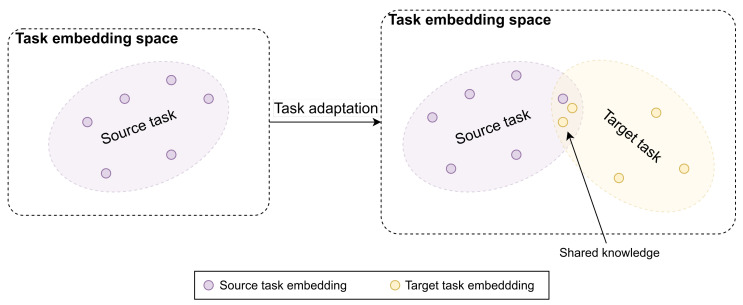
An illustration of the task embedding space. Purple and yellow regions denote the knowledge learned from the source and target tasks, respectively. Applying the proposed task adaptation algorithm will lead to the expansion of the task embedding space due to the acquisition of the knowledge of the target task. In addition, the intersection between those two regions indicates the shared common knowledge between the two tasks.

Accordingly, the proposed method is two-fold. Firstly, an adaptation algorithm is proposed to allow the agent to learn the new target task by expanding its knowledge. More concretely, on top of the knowledge that the agent has learned from a source task, the knowledge of a target task is added. In addition, the agent repeatedly uses such knowledge to learn the target task and review the previously learned source task to ensure that the learning performance on the target task is high, while the deterioration of the learning performance on the source task is small. Secondly, to support the expansion of the to-be-learned knowledge, a novel imitation learning (IL) agent is proposed. This agent encodes the learned knowledge into a latent space, namely task embedding space, in which the learned knowledge from task *x* at time step *t* can be represented by a high-dimensional vector $z_{x}^{t}\in R^{n}$. Figure 1 illustrates the task embedding space before and after applying the proposed task adaptation algorithm. The task embedding space allows the proposed adaptation algorithm to add the new knowledge of the target task while minimizing its impacts on the source task’s knowledge. In addition, since the source and target tasks are related to each other, there are some common knowledge between those two tasks. This shared common knowledge can be captured by the task embedding that helps accelerate the adaptation process. The details of the proposed method are provided in the following sub-sections.

### 4.1. The Proposed Agent

**Figure 2:**
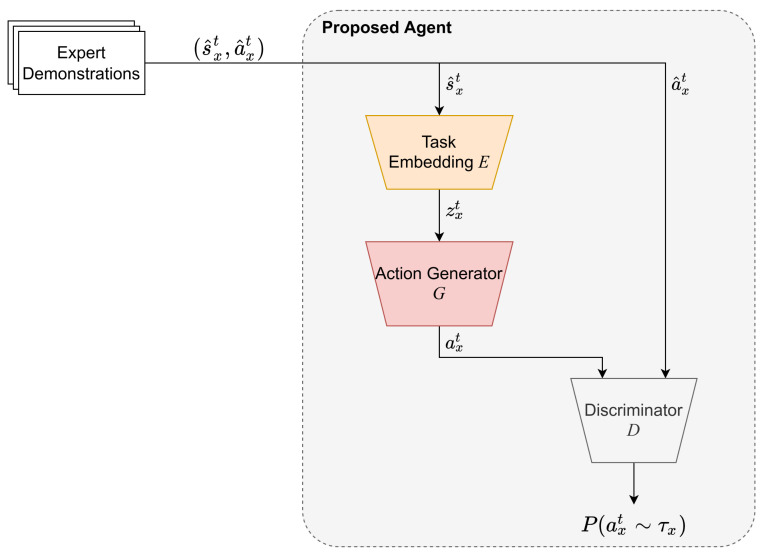
The neural network architecture of the proposed agent.

In this subsection, the proposed agent is described in detail. The proposed agent is an imitation learning method that finds an optimal policy for the source task using expert-generated demonstration data. The agent is capable of encoding the learned knowledge into a task embedding in order to support the later adaptation progress. The architecture of the proposed agent is illustrated in Figure 2. The proposed agent is a combination of three deep feed-forward networks *E*, *G*, and *D*, which have different responsibilities.

#### 4.1.1. Task-Embedding Network *E*

The task-embedding network *E* is designed to encode the learned knowledge into a high-dimensional task embedding space. Specifically, *E* maps a state $s_{x}^{t}$ of task *x* at time step *t* into a task embedding $z_{x}^{t}=E\left(s_{x}^{t}\right)$, $z_{x}^{t}\in R^{n}$. Since $z_{x}^{t}$ contains the information of the task, it is expected that $z_{x}^{t}$ can capture the similarities and differences between source and target tasks. In order to achieve that, contrastive learning is introduced to train *E*. Contrastive learning aims to bring task embeddings of the same task close to each other in the task embedding space and to push dissimilar ones far apart. In order words, *E* is trained to minimize distance $d(z_{S}^{t},z_{S}^{t})$ and maximize distance $d(z_{S}^{t},z_{T}^{t})$, where d(·) is a negative cosine similarity function defined as

(2) $$d\left(z_{x}^{t},z_{y}^{t}\right)=-\frac{z_{x}^{t}\cdot z_{y}^{t}}{\left|\right|z_{x}^{t}\left||*|\right|z_{y}^{t}\left|\right|}$$

where *x* and *y* can be the same or different task.

The optimization function $L_{E}$ to train *E* is defined as follows:

(3) $$\min_{E}L_{E}\left(z_{x}^{t},z_{y}^{t}\right)=𝟙\left[x=y\right]d\left(z_{x}^{t},z_{y}^{t}\right)+𝟙\left[x\neq y\right]\left(-d\left(z_{x}^{t},z_{y}^{t}\right)\right)$$

where 𝟙(·)∈{0,1} is an indicator function.

#### 4.1.2. Action Generator Network *G* and Discriminator Network *D*

The action generator network *G* aims to generate an optimal action $a_{x}^{t}$ using the input task embedding $z_{x}^{t}$. The discriminator network *D* is designed to distinguish between expert action ${\hat{a}}_{x}^{t}$ and the training agent’s action $a_{x}^{t}$. The intuition behind this is that the expert actions are assumed to be optimal in the imitation learning setting, thus, *G* are trained to minimize the difference between ${\hat{a}}_{x}^{t}$ and $a_{x}^{t}$. In order to achieve that, the adversarial loss [57] is applied for both networks:

(4) $$\min_{G}\max_{D}L_{GD}\left({\hat{a}}_{x}^{t},a_{x}^{t}\right)=E\left[logD\left(a_{x}^{t}\right)\right]+E\left[log\left(1-D\left({\hat{a}}_{x}^{t}\right)\right)\right]$$

The optimal policy is achieved using a RL-based policy gradient method, which relies on reward signal $r=-logD({\hat{a}}_{x}^{t})$ provided by the discriminator.

#### 4.1.3. Full Objective

During the source task’s learning process, a set of expert-generated demonstrations $\left\{\tau_{S}^{1},\tau_{S}^{2},\ldots \right\}$ is provided where each demonstration is a sequence of state-actions pairs $\tau_{S}^{i}=\left\{\left({\hat{s}}_{S}^{t},{\hat{a}}_{S}^{t}\right),\ldots \right\}$. The task embedding for each demonstration state $z_{S}^{t}$ at time step *t* can be computed using $z_{S}^{t}=E\left({\hat{s}}_{S}^{t}\right)$. It should be noted that the contrastive loss function $L_{E}$ used to train *E* requires two inputs $z_{x}^{t}$ and $z_{y}^{t}$, where *x* and *y* can be of the same or different task. In this source task learning process, the target task demonstrations are not provided yet, thus, the second task embedding input $z_{S}^{\prime t}$ is generated by introducing the Gaussian noise μ∼N(0,1) to augment ${\hat{s}}_{x}^{t}$ as follows:

(5) $$z_{S}^{\prime t}=E\left({\hat{s}}_{S}^{\prime t}\right)$$

where ${\hat{s}}_{S}^{\prime t}={\hat{s}}_{S}^{t}+\mu$. In addition, since ${\hat{s}}_{S}^{\prime t}$ is an augmentation of ${\hat{s}}_{S}^{t}$, it might not belong to the state space $S_{S}$ of the source task. Thus, the resulting $z_{S}^{\prime t}$ is not used as an input to *G* to generate an action, but it is used to help compute the loss $L_{E}$ only. This means that $z_{S}^{\prime t}$ can be treated as a constant. In other words, the gradient flows back from $z_{S}^{\prime t}$ is unnecessary in the backpropagation. This can be indicated using the stop-gradient operation stopgrad(·) as follows [58,59]:

(6) $$z_{S}^{\prime t}=stopgrad\left(E\left({\hat{s}}_{S}^{\prime t}\right)\right)$$

With the generated action $a_{S}^{t}=G\left(z_{S}^{t}\right)$, the full objective function to train the proposed agent on the source task is

(7) $$\min_{E,G}\max_{D}L=L_{E}\left(z_{S}^{t},z_{S}^{\prime t}\right)+L_{GD}\left({\hat{a}}_{S}^{t},a_{S}^{t}\right)$$

The algorithm to train the proposed agent on the source task is outlined in Algorithm 1.
**Algorithm 1** Training the proposed agent on the source task.1:**Input**2:  $\left\{\tau_{S}^{1},\tau_{S}^{2},\ldots \right\}$ A set of expert demonstrations on the source task3:Randomly initialize task embedding network *E*, generator *G* and discriminator *D*4:**for***k* = 0, 1, 2, … **do**5:   Sample an expert demonstration $\tau_{S}^{i}$6:   Sample state-action pairs $\left({\hat{s}}_{S}^{t},{\hat{a}}_{S}^{t}\right)$∼$\tau_{S}^{i}$7:   Compute $z_{S}^{t}=E\left({\hat{s}}_{S}^{t}\right)$8:   Compute $z_{S}^{\prime t}=stopgrad\left(E\left({\hat{s}}_{S}^{t}+\mu \right)\right)$9:   Generate action $a_{S}^{t}=G\left(z_{S}^{t}\right)$10: Compute the loss $L=L_{E}\left(z_{S}^{t},z_{S}^{\prime t}\right)+L_{GD}\left({\hat{a}}_{S}^{t},a_{S}^{t}\right)$11: Update the parameters of *F*, *G*, and *D*12: Update policy $\pi_{S}$ with the reward signal $r=-logD({\hat{a}}_{S}^{t})$13:**end for**14:**Output**15: $\pi_{S}$    Learned policy for source task

### 4.2. The Proposed Task Adaptation Algorithm

**Figure 3:**
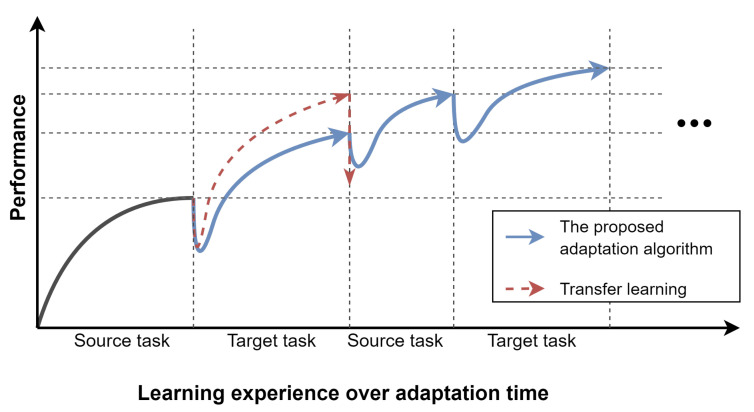
An illustration of the performance of an agent on the source and target tasks over adaptation time.

Leveraging the task embedding space learned by the proposed agent, a novel adaptation algorithm is presented in order to adapt the agent to a new target task by adding the knowledge of the target task to the task-embedding space as shown in Figure 2. In addition, to prevent losing the previously learned knowledge to perform the source task, a novel idea based on repetition learning is applied in the proposed adaptation algorithm. The idea can be illustrated as shown in Figure 3. The intuition behind this idea is that during the adaptation process, the agent is allowed to repeatedly review how to perform the previously learned source task while learning the target task. Each time the agent switches to a different task, its performance drops, but then it recovers. This distinctive learning process allows the agent to continuously review its learned knowledge and generalize to both source and target tasks, resulting in an agent that can perform well on both tasks. It is similar to humans; when humans repeatedly practice an action, it leads to better performance. In addition, the process enables the agent to surpass the performance of an agent that is adapted using transfer learning. As shown in Figure 3, using transfer learning, the adapted agent completes its adaptation process right after adapting the source task to the target task. For this reason, when facing the source task again after adaptation, the performance of the agent deteriorates due to the catastrophic forgetting problem.

It is important to note that, theoretically, the more knowledge the agent gains, the higher performance the agent can provide on both source and target tasks. As shown in Figure 3, after facing the source task again, the performance of the agent on the source task increases. However, in practice, there is still an amount of performance deterioration on the source task since the agent is not able to fully utilize the learned knowledge. This observation is further discussed in the evaluation and discussion sections.

In this paper, a hyperparameter λ∈[0,1] is introduced, which denotes the probability that the agent repeatedly reviews the source task’s knowledge. With λ, the balance between the performance on the target task and the performance deterioration on the source task can be controlled. For instance, the higher the value of λ, the higher the probability that the agent can review the previously learned source task, resulting in a smaller deterioration of the source task’s performance in exchange for low performance on the target task. It should be noted that if λ≈0, the proposed task adaptation algorithm can be seen as a transfer learning method where it is only focused on improving the target task’s performance. The task adaptation algorithm is outlined in Algorithm 2.
**Algorithm 2** The proposed adaptation algorithm.1:**Input**2:  $\left\{\tau_{T}^{1},\tau_{T}^{2},\ldots \right\}$ A set of expert demonstrations on the target task3:  $\left\{\tau_{S}^{1},\tau_{S}^{2},\ldots \right\}$ A set of expert demonstrations on the source task4:Randomly initialize task embedding network *E*, generator *G* and discriminator *D*5:**for***k* = 0, 1, 2, … **do**6:  Sample an expert demonstration on the target task $\tau_{T}^{i}$7:  Sample an expert demonstration on the source task $\tau_{S}^{i}$8:  Sample state-action pairs $\left({\hat{s}}_{S}^{t},{\hat{a}}_{S}^{t}\right)$∼$\tau_{S}^{i}$ and $\left({\hat{s}}_{T}^{t},{\hat{a}}_{T}^{t}\right)$∼$\tau_{T}^{i}$9:  *n* ← uniform random number between 0 and 110:   **if** n<λ **then**           ▹ Review source task’s learned knowledge11:    Compute $z_{S}^{t}=E\left({\hat{s}}_{S}^{t}\right)$12:    Compute $z_{T}^{t}=stopgrad\left(E\left({\hat{s}}_{T}^{t}\right)\right)$13:    Generate action $a_{S}^{t}=G\left(z_{S}^{t}\right)$14:    Compute the loss $L=L_{E}\left(z_{S}^{t},z_{T}^{t}\right)+L_{GD}\left({\hat{a}}_{S}^{t},a_{S}^{t}\right)$15:  **else**                           ▹ Learn target task16:    Compute $z_{T}^{t}=E\left({\hat{s}}_{T}^{t}\right)$17:    Compute $z_{S}^{t}=stopgrad\left(E\left({\hat{s}}_{S}^{t}\right)\right)$18:    Generate action $a_{T}^{t}=G\left(z_{T}^{t}\right)$19:    Compute the loss $L=L_{E}\left(z_{T}^{t},z_{S}^{t}\right)+L_{GD}\left({\hat{a}}_{T}^{t},a_{T}^{t}\right)$20:  **end if**21:  Update the parameters of *F*, *G*, and *D*22:  Update policy $\pi_{S}$ with the reward signal $r=-logD({\hat{a}}_{S}^{t})$23:**end for**24:**Output**25:  $\pi_{ST}$    Learned policy for both source and target task

## 5. Performance Evaluation

In this section, the performance of the proposed method is evaluated in comparison with baselines. To support the evaluation, different simulated tasks with varying difficulty levels ranging from simple to complex ones were utilized. The details of these tasks are described in the next subsection. A set of experiments are designed in order to answer the following essential questions:

- Can the proposed IL agent provide a competitive performance on the source task?
- Can the adaptation algorithm enable the agent to adapt its learned knowledge to the target task in order to outperform the baselines?
- By leveraging the repetition learning to expand the agent’s knowledge, can the adaptation algorithm reduce the deterioration of the agent’s performance on the source task?

### 5.1. Experimental Settings

#### 5.1.1. Simulated Tasks

**Figure 4:**
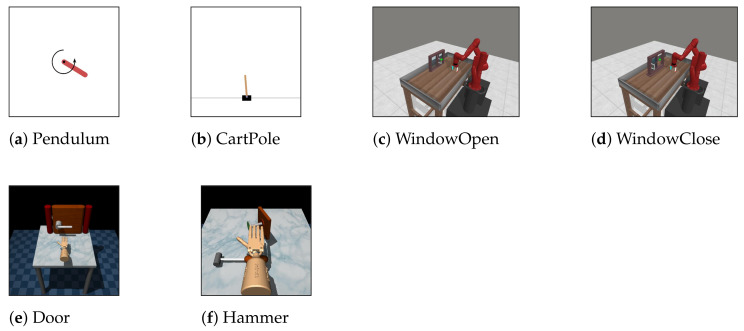
Visual rendering of five simulated tasks used in the experiment.

In order to examine the effectiveness of the proposed method, six simulated tasks with varying difficulties were considered: Pendulum [60], CartPole [60,61], WindowOpen [62], WindowClose [62], Door [63], and Hammer [63]. The task difficulty is varied along two axes; the size of the state space and the size of the action space. The detailed descriptions and visualizations of these tasks are shown in Table 1 and Figure 4. From such tasks, three experiments were conducted, each of which included two different tasks—a source task and a target task. The detailed descriptions of these experiments are shown in Table 2.

In order to train and adapt the proposed IL agent, expert demonstrations for both source and target tasks must be provided. In this experiment, the proximal policy optimization (PPO) method was chosen to be trained on each task in order to create an expert RL agent. The reason behind this decision was that PPO was recently showing the best result for many complex tasks. After that, the demonstrations were collected by executing the trained PPO expert agent in the simulated task. For the source task, 30 demonstrations were collected to provide sufficient data for training the proposed agent [57]. In the adaptation process, the proposed agent already learned the knowledge of the source task, thus, a smaller number of demonstrations for the target task is required. Therefore, only 15 demonstrations were collected for the target task.

#### 5.1.2. Baselines

To evaluate the performance of the proposed method, a number of baselines were considered. Firstly, to assess the performance of the proposed agent on a source task, two RL baselines were used, which are proximal policy optimization (PPO) [64] and neural fitted Q-iteration (NFQI) [65]. PPO is a policy gradient method, while NFQI is a value-based method that tries to estimate the Q-function using a deep feed-forward network. Secondly, after training the agent on the source task, the proposed adaptation algorithm was applied in order to adapt the trained agent to a new target task. The performance of the agent after adaptation was evaluated through the comparison with transfer learning-based baselines, which are fine-tuning and TA-TL [66]. Fine-tuning is a common transfer learning technique that simply re-trains the agent on a new target task. Fine-tuning was applied to both the proposed agent and PPO, resulting in two baselines for the evaluation. Meanwhile, TA-TL is a policy adaptation method, where first it utilizes the NFQI agent to find an optimal policy on a source task, then that policy is transferred to a new target task. In order to provide a fair comparison, each baseline was evaluated for 100 trials. The success rate and average cumulative reward were used as performance metrics. The success rate indicates the percentage of trials in which the baseline can successfully complete a task. The average cumulative reward measures how well the baseline performed in a trial.

#### 5.1.3. Implementation and Training Details

In order to perform the experiments, a personal computer running Ubuntu 20.04 with an Intel i7-8750H @ 2.20GHz, 16 GB RAM, and NVIDIA GTX 1080 Ti was used. PyTorch [67] and Tianshou [68] were utilized as deep learning frameworks to implement the proposed adaptation method and baselines. Adam optimizer with an initial learning rate of $10^{-4}$ was used for training the proposed agent. The dimension *n* of the task embedding $z_{x}^{t}$ and the value of λ were set to 64 and 0.1, respectively.

### 5.2. Results

In this subsection, the evaluation results of the proposed agent and adaptation algorithm are presented to highlight their effectiveness in tackling the task adaptation problem in imitation learning.

#### 5.2.1. Performance of the Proposed Agent on the Source Task

**Figure 5:**
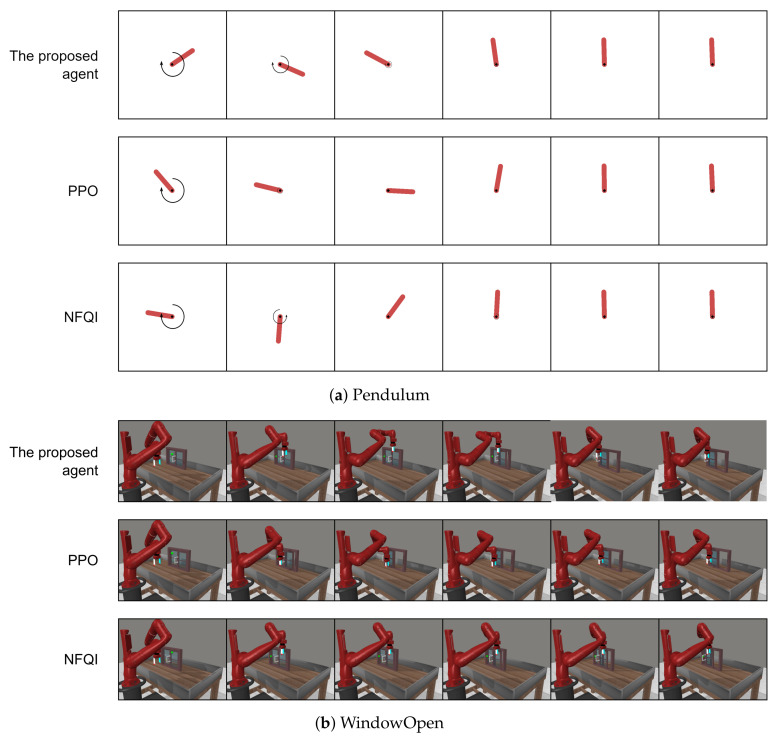

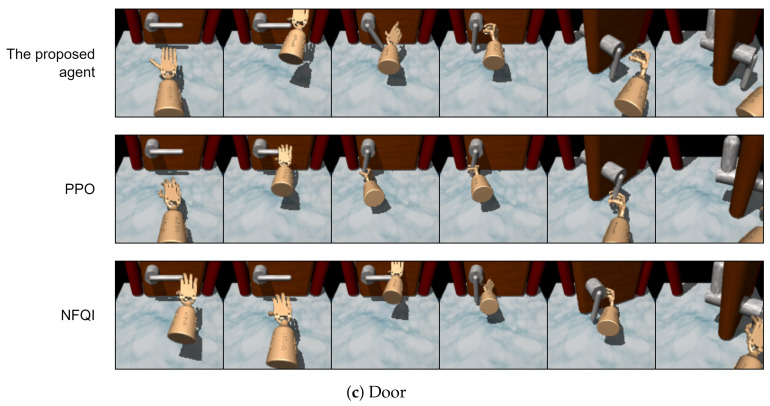
A visualization of the behavior of the proposed agent and baselines on source tasks.

Table 3 reports the performance of the proposed agent on the source tasks (i.e., Pendulum, WindowOpen, and Door) against two RL baselines: PPO and NFQI. In addition, Figure 5 visualizes their behaviors when performing the source tasks. It can be observed that the proposed agent and two baselines could accomplish source tasks by keeping the pendulum vertical (Figure 5a), successfully opening the window and the door (Figure 5b,c). The proposed imitation learning agent was able to produce relatively similar behaviors to PPO. This result demonstrated that the proposed agent was trained successfully in order to imitate the expert behaviors. Table 3 shows that PPO always provided the best performance in terms of success rate and average cumulative reward on three different source tasks. This result was reasonable since PPO is a reinforcement learning method, thus, it has a direct access to the task environment, including states and the reward signal. On the other hand, the proposed agent is an imitation learning method that learns to perform the task using only expert demonstrations. Despite that disadvantage, the proposed agent could consistently perform well on all source tasks with varying difficulties and almost achieved similarly high performance to PPO. It should be noted that the performance of all agents always decreased when being tested on a more complicated task with more extensive state and action spaces, especially the Door task. However, the reduction in performance between the proposed agent and PPO was comparable. On the other hand, there was a significant gap between the proposed agent and the NFQI performance. The NFQI agent showed the largest reduction in terms of success rate, i.e., from 100% success rate on the simple Pendulum task to only 65% on the challenging Door task. This was because the Q-function approximation in NFQI did not work well with the task with large state and action spaces [65]. In summary, the results showed that the proposed agent could provide relatively high and consistent performance that is close to the expert PPO on different source tasks with various difficulty levels.

#### 5.2.2. Performance of the Proposed Agent on the Target Task after Adaptation

**Figure 6:**
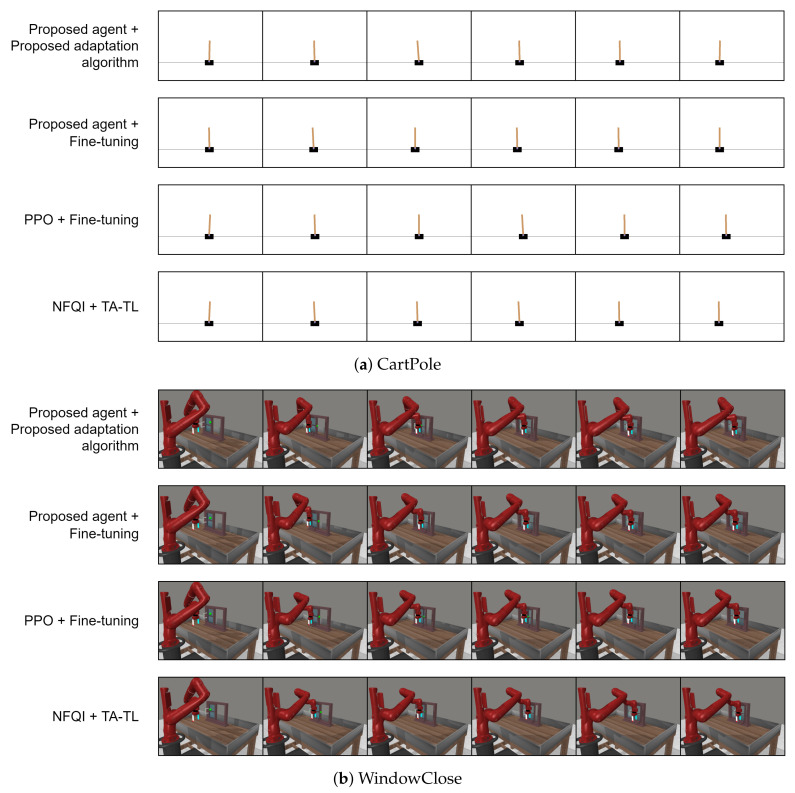

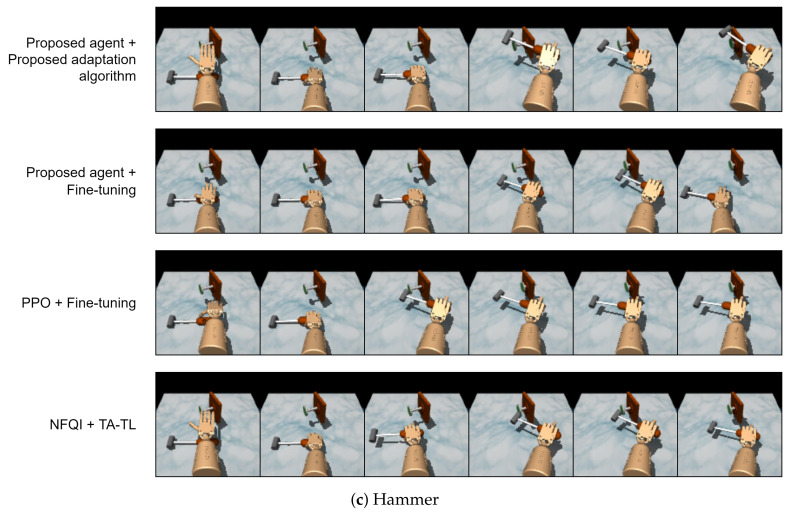
A visualization of the behavior of the proposed agent and baselines on target tasks.

All agents trained on the source task were adapted to the target task in order to evaluate the performance of the proposed adaptation algorithm in comparison with other transfer learning baselines. The result is tabulated in Table 4. The behavior of those agents when performing target tasks is visualized in Figure 6. It can be seen that the proposed adaptation method and baselines provide comparably similar behaviors in order to solve target tasks. This result indicated that the proposed method successfully adapted and transferred the agent’s knowledge to the new target task. Moreover, it can be observed from Table 4 that the proposed method, which is a two-fold method, including the proposed agent and the adaptation algorithm, outperformed other transfer learning-based baselines. In addition, it performed highly well and consistently on the complex WindowClose and Hammer tasks. On the other hand, applying fine tuning to the proposed agent led to a significant reduction in the adapted agent’s performance, especially on the complex Hammer task which achieved only a 50% success rate. Moreover, its performance was the lowest compared to other transfer learning baselines. This indicated that the trained agent on the source task (i.e., Door) failed to transfer its learned knowledge to the target task (i.e., Hammer). The reason could be because the adapted agent using fine tuning failed to learn state and action mappings from the source to the target task due to the size of the state and action spaces of those two tasks being different as shown in Table 1. This observation indicates that fine tuning was not suitable for the proposed agent. On the other hand, applying fine tuning to the PPO agent provided a consistent performance across all three tasks. At the same time, applying TA-TL to the NFQI agent was not able to produce a high success rate due to the high complexity of the WindowClose and Hammer tasks.

The results demonstrated that the proposed method not only outperformed baselines in terms of success rate on all target tasks, but notably produced a consistently high performance, even on the most difficult task. This proved the potential of the proposed method in order to tackle the task adaptation problem in imitation learning. However, it should be noted that the research objective is not only to achieve high performance on the target task, but also to avoid the performance deterioration on the source task. Therefore, the performance of the adapted agent on source tasks will be assessed next in order to evaluate the decline of the agent’s performance after adaptation.

#### 5.2.3. Performance of the Proposed Agent on the Source Task after Adaptation

Table 5 shows the deterioration in success rate of the adapted agent on source tasks compared to the one before the adaptation. The lower value of the deterioration illustrates a better result. It can be observed that as the difficulty level of the target task increased, the deterioration became more notable. In addition, three baselines were not able to maintain high performance on the source task. Even on the simple Pendulum task, the deterioration was extremely high compared to the proposed adaptation algorithm. This was due to the fact that those transfer learning baselines were designed to optimize the performance of the agent only on the target task. Thus, it was obvious that the performance of those adapted agents dropped significantly on the source task. On the other hand, the deterioration of the proposed method was the lowest compared to other baselines, which indicated that the proposed adaptation algorithm successfully retained the learned knowledge from the source tasks and reduced the negative effect of catastrophic forgetting.

#### 5.2.4. Computational Complexity

Besides evaluating the performance of the proposed task adaptation method in terms of success rate, its computational cost was also assessed in order to provide an adequate study of its overall performance. Table 6 shows the training time required to adapt a trained agent to a new target task in each experiment. It can be observed that the training time of the proposed adaptation method was slightly better than the training time when applying fine tuning to PPO, especially on two complex WindowOpen-WindowClose and Door–Hammer experiments. On the other hand, compared to TA-TL, the proposed adaptation method required a higher training time on all three experiments. This result was expected since, during the proposed adaptation process, the agent had to not only learn the new task, but also review the previously learned source task. However, it should be noted that the training time of the proposed adaptation method can be further improved by leveraging the parallel training process [68,69].

## 6. Discussion

In this section, the effects of applying repetition learning on the performance of the proposed method and the important role of the task embedding network *E* are discussed in detail.

The experimental results assessed in the previous section have shown the potential of the proposed adaptation method in tackling the task adaptation problem in imitation learning. As shown in Table 3 and Table 4, the proposed method could provide consistent and high performance in terms of success rate and average cumulative reward on both source and target tasks with varying difficulty levels. This indicates that the proposed method can be applied to more challenging tasks with larger state and action spaces. Moreover, Table 5 shows that the performance deterioration on the source task was also reduced compared to transfer learning baselines. This promising result demonstrates the effectiveness of the proposed adaptation method, in which the idea of repetition learning was leveraged in order to allow the agent to review the previously learned source task. Although the success rate and training time remained limited, the proposed method presents a plausible approach to tackle the task adaptation problem in imitation learning. It can be further improved in order to provide better overall performance toward practical imitation learning tasks.

**Figure 7:**
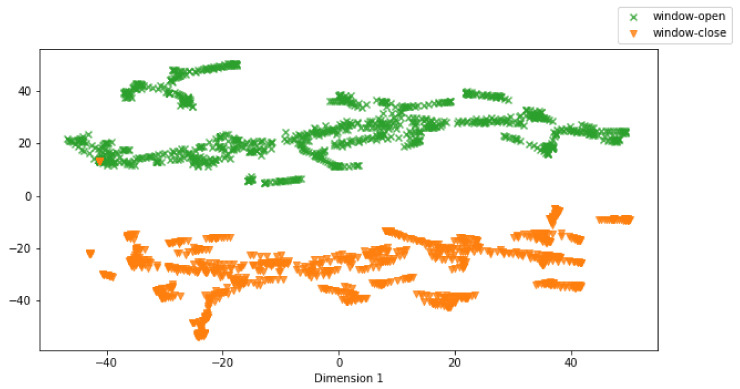
Visualization of clustering results on task embedding vectors $z_{S}^{t}$ and $z_{T}^{t}$. Different colors mark different tasks.

In order to support the idea of repetition learning, an imitation learning agent was proposed, which was able to encode its learned knowledge into a task-embedding space. To provide an ablation study of the task embedding network *E* in the proposed agent, a small experiment was conducted, where a number of task embeddings $z_{S}^{t}$ and $z_{T}^{t}$ were collected by executing the adapted agent in the WindowOpen–WindowClose experiment on both source task (i.e., WindowOpen) and target task (i.e., WindowClose). The WindowOpen–WindowClose was chosen because both source and target tasks are similar and have a large and equal size of the state space, which can provide a sufficient ablation result. In each task, the adapted agent was run in the simulation over 100 trials. After that, t-distributed stochastic neighbor embedding (t-SNE) was applied in order to project the collected high-dimensional task embeddings to a two-dimensional space for visualization as shown in Figure 7. t-SNE captures the distance relation between task embeddings. If two embeddings were close in the task-embedding space, they stay close in the resulting visualization, and vice versa. Therefore, from Figure 7, it can be seen that task embeddings of the source and target tasks were well separated. Moreover, Figure 7 also shows that some target task embeddings were mixed with the source task embeddings. This was expected since the WindowOpen and WindowClose tasks shared the same structure (i.e., robot hand and window), thus, these target task embeddings represented the shared knowledge between the source and target tasks. This result indicates that the proposed adaptation method not only successfully expands the task embedding space without forgetting the previously learned knowledge, but also leverages the source task’s knowledge in order to accelerate and adapt to the new target task. This leads to high performance on the target task shown in Table 4 and a low performance deterioration on the source task shown in Table 5.

Although the novel idea of applying repetition learning and encoding the task knowledge into a task embedding has significantly improved the adapted agent on both tasks, there is still one limitation. As shown in Figure 3, ideally, the adapted agent should be able to perform both source and target tasks better over time and eventually surpass its performance on the source task before being adapted. However, as shown in the experimental results, there was an amount of deterioration in the source task’s performance, thus, the proposed method is still limited compared to human learning ability. Overcoming this problem can be served as a key step toward building a continual learning agent, where the agent can learn and adapt to not only one but multiple target tasks. In future work, this will be the main focus of the authors in order to provide a general-purpose agent that can become a better learner over time, i.e., learning new tasks better and faster, and performing better on previously learned tasks.

## 7. Conclusions

In this paper, a novel task adaptation method for imitation learning was proposed. The proposed adaptation method leverages the idea of repetition learning in neuroscience allowing the agent to repeatedly review the previously learned source task while learning a new target task. The experimental results on simulated tasks with varying difficulties show that the proposed method is able to consistently provide high performance on the target task and minimizes the deterioration of the source task’s performance. Moreover, it demonstrates the effectiveness of the proposed method compared to transfer learning in enabling the agent to expand its knowledge without forgetting the knowledge learned from the source task, resulting in an adapted agent that is able to perform well on both tasks. Despite some limitations in the success rate and computational cost, the results indicate the potential of the proposed method to be applied in practical imitation learning tasks.

## Figures and Tables

**Table 1:** Description of six simulated tasks used in the experiment.

Task	Size of State Space	Size of Action Space	Difficulty Level	Description
Pendulum [60]	3 (continuous)	1 (continuous)	Easy	Swinging up a pendulum.
CartPole [60,61]	4 (continuous)	1 (continuous)	Easy	Preventing the pendulum from falling over by applying a force to the cart.
WindowOpen [62]	39 (continuous)	4 (continuous)	Medium	Opening a window.
WindowClose [62]	39 (continuous)	4 (continuous)	Medium	Closing a window.
Door [63]	39 (continuous)	28 (continuous)	Hard	A 24-DoF hand attempts to undo the latch and swing the door open.
Hammer [63]	46 (continuous)	26 (continuous)	Hard	A 24-DoF hand attempts to use a hammer to drive the nail into the board.

**Table 2:** Description of three experiments conducted to evaluate the performance of the proposed method.

Experiment	Source Task	Target Task	Difficulty Level	Description
Pendulum–CartPole	Pendulum	CartPole	Easy	A simple experiment in which both source and target tasks have small state and action spaces.
WindowOpen–WindowClose	WindowOpen	WindowClose	Medium	Both source and target tasks have a large state space but small action space.
Door–Hammer	Door	Human	Hard	A challenging experiment in which both source and target tasks have large state and action spaces.

**Table 3:** The performance of the proposed agent on source tasks.

		Pendulum	WindowOpen	Door
Success rate	Proposed agent	100%	94%	87%
	PPO [64]	100%	97%	91%
	NFQI [65]	100%	76%	65%
Average cumulative reward	Proposed agent	−146.51 ± 85.24	1586.38 ± 229.00	2250.04 ± 1428.60
	PPO [64]	−134.77 ± 93.59	1827.56 ± 410.98	2450.42 ± 1303.48
	NFQI [65]	−189.01 ± 87.09	752.00 ± 476.77	1252.55 ± 1213.15

**Table 4:** The performance of the proposed agent on target tasks after adaptation.

		CartPole	WindowClose	Hammer
Success rate	Proposed agent + Proposed adaptation algorithm	100%	83%	82%
	Proposed agent + Fine-tuning	77%	72%	50%
	PPO [64] + Fine-tuning	87%	80%	77%
	NFQI + TA-TL [66]	80%	63%	67%
Average cumulative reward	Proposed agent + Proposed adaptation algorithm	500.00±0.0	2340.59±642.69	13,137.42 ± 2709.57
	Proposed agent + Fine-tuning	433.44±86.52	1513.07±566.09	1741.76±1035.17
	PPO [64] + Fine-tuning	487.63±32.74	2215.98±608.33	3022.64±1115.92
	NFQI + TA-TL [66]	476.63±61.84	1447.53±641.16	2591.46±1231.70

**Table 5:** The performance of the proposed agent on source tasks after adaptation. These scores represent the deterioration in success rate compared to the one before the adaptation.

	Pendulum	WindowOpen	Door
Proposed agent + Proposed adaptation algorithm	18%	32%	44%
Proposed agent + Fine-Tuning	41%	73%	74%
PPO [64] + Fine-tuning	32%	58%	83%
NFQI + TA-TL [66]	24%	62%	51%

**Table 6:** The training time (s/epoch) of the proposed task adaptation algorithm.

	Pendulum–CartPole	WindowOpen–WindowClose	Door–Hammer
Proposed agent + Proposed adaptation algorithm	87.051	163.768	503.19
Proposed agent + Fine-tuning	74.680	114.290	321.87
PPO [64] + Fine-tuning	86.801	184.472	557.416
NFQI + TA-TL [66]	58.499	121.510	352.53

## Data Availability

Not applicable.
